# Functional analysis of mammalian phospholipase D enzymes

**DOI:** 10.1042/BSR20181690

**Published:** 2018-12-07

**Authors:** Aniruddha Panda, Rajan Thakur, Harini Krishnan, Amruta Naik, Dhananjay Shinde, Padinjat Raghu

**Affiliations:** 1 National Centre for Biological Sciences-TIFR, GKVK Campus, Bellary Road, Bangalore 560065, India; 2 Manipal University, Madhav Nagar, Manipal 576104, Karnataka, India

**Keywords:** Drosophila, lipids, membranes, mass spectrometry, phospholipase D

## Abstract

Phosphatidylcholine (PC)-specific phospholipase D (PLD) hydrolyzes the phosphodiester bond of the PC to generate phosphatidic acid (PA) and regulates several subcellular functions. Mammalian genomes contain two genes encoding distinct isoforms of PLD in contrast with invertebrate genomes that include a single PLD gene. However, the significance of two genes within a genome encoding the same biochemical activity remains unclear. Recently, loss of function in the only PLD gene in *Drosophila* was reported to result in reduced PA levels and a PA-dependent collapse of the photoreceptor plasma membrane due to defects in vesicular transport. Phylogenetic analysis reveals that human PLD1 (hPLD1) is evolutionarily closer to dPLD than human PLD2 (hPLD2). In the present study, we expressed hPLD1 and hPLD2 in *Drosophila* and found that while reconstitution of hPLD1 is able to completely rescue retinal degeneration in a loss of function dPLD mutant, hPLD2 was less effective in its ability to mediate a rescue. Using a newly developed analytical method, we determined the acyl chain composition of PA species produced by each enzyme. While dPLD was able to restore the levels of most PA species in *dPLD^3.1^* cells, hPLD1 and hPLD2 each were unable to restore the levels of a subset of unique species of PA. Finally, we found that in contrast with hPLD2, dPLD and hPLD1 are uniquely distributed to the subplasma membrane region in photoreceptors. In summary, hPLD1 likely represents the ancestral PLD in mammalian genomes while hPLD2 represents neofunctionalization to generate PA at distinct subcellular membranes.

## Introduction

Phospholipases are enzymes whose activity can remodel the structure of glycerophospholipids and generate products that can serve as second messengers. Based on the position of the phosphodiester bond at which they act, several different classes of phospholipases have been described. For example, receptor-activated phospholipase C enzymes at the plasma membrane can hydrolyze the lipid phosphatidylinositol 4,5 bisphosphate (PIP_2_) to generate inositol 1,4,5 trisphosphate (IP_3_) and diacylglycerol (DAG) [1]. Phosphatidylcholine (PC)-specific phospholipase D (PLD) is an enzyme that hydrolyzes PC to generate phosphatidic acid (PA). PA binds to a large and diverse group of proteins [2] and it is thought that modulation of their activities by PA is a principal mediator of the biological effects of PLD. PA generated by PLD has been reported to regulate several subcellular processes including vesicular transport [3–7], mTOR and S6K activity [8], and NADPH oxidase activity [9]. A large number of genes encoding PLD enzymes have been described in a range of genomes including those from viral, prokaryotic, and eukaryotic organisms [10]. A subset of these contain a HxKxxxxDx6 GSxN motif (HKD) that is responsible for enzymatic activity. While this motif itself includes other enzymes that catalyze the same chemical reaction (such as nucleases/end joining enzymes), the presence of additional domains such as the PX and PH domains defines a family of so-called conventional PLD enzymes in eukaryotic genomes. The genomes of simpler eukaryotes encodes a single conventional PLD but those of complex metazoans contain multiple genes that encode conventional PLD like proteins; for example most mammalian genomes contain PLD1 and PLD2 gene [10]. Molecular cloning and biochemical analysis have provided detailed information of the biochemical activity of PLD1 and PLD2, their precise cell-biological function remain unclear. Studies on gene knockouts of PLD1 and PLD2 in mouse have revealed that knockouts of mouse PLD1 and PLD2 result in homozygous viable adults with no gross defects (reviewed in [11]). Analysis of these knockouts has described a number of subtle and specific phenotypes in relation to tumor biology [12,13], neurobiology [14–17], platelet function [18,19] thus implicating these enzymes in relevant physiological process. However, the cell biological significance of evolving two genes encoding the same biochemical activity remains a topic of investigation.

The function of PA as a mediator of vesicular transport has been widely studied. Initial insights into its role in controlling vesicular transport arose from the observation that *in vitro*, Arf proteins, key regulators of vesicular transport stimulate mammalian PLD activity [20,21]. Several studies, primarily involving PLD overexpression have reported effects on vesicular transport in cultured mammalian cells [3–6]. However, studies using PLD1 and PLD2 knockout mice are yet to map effects on vesicular transport seen on PLD overexpression in cultured mammalian cells to cell biological processes *in vivo*. Loss of PLD in *Caenorhabditis elegans* is reported to result in phenotypes related to lifespan and metabolism [22,23] and manipulation of PLD1 in zebrafish is reported to result in intersegmental vascular pattern defects [24]. However, in these models of PLD depletion, the role of altered vesicular transport in the reported phenotypes is not known. Notable exceptions to this are loss-of-function mutants for PLD in yeast and *Drosophila*; in both these organisms the genome includes a single PLD gene. In yeast, mutants of PLD (SPO14) are viable under vegetative conditions but result in defective sporulation during nitrogen deprivation [25–27] and the role of PA binding to SPO20, a v-SNARE in regulating vesicular transport to the bud has been elucidated [28]. Most recently, loss of the only PLD in *Drosophila* has been reported to result in collapse of the apical plasma membrane in photoreceptors during illumination coupled with a defect in the recycling of vesicles to the apical plasma membrane following light-induced endocytosis. Photoreceptors contain a light-activated PLD and the phenotypes of dPLD loss of function were correlated with the levels of PA in these cells [29]. The availability of a null mutant in the only PLD gene in *Drosophila* with a clear phenotype related to vesicular transport offers an opportunity to test the function of conventional human PLD1 (hPLD1) and human PLD2 (hPLD2) *in vivo*. In the present study, we report that hPLD1 but not hPLD2 is able to fully support photoreceptor structure during illumination.

## Experimental procedures

### Fly cultures and stocks

Flies were reared on medium containing corn flour, sugar, yeast powder, and agar along with antibacterial and antifungal agents. Flies were maintained at 25°C and 50% relative humidity. There was no internal illumination within the incubator and the flies were subjected to light pulses of short duration only when the incubator door was opened. When required, flies were grown in an incubator with constant illumination from a white light source (intensity ∼2000 lux). The wild-type used for all experiments was Red Oregon-R. The expression of transgenes was controlled using the bipartite GAL4-UAS system [30] where spatial and temporal expression are controlled by suitable enhancers (e.g. Rhodopsin 1, heat shock enhancer) that express GAL4 in the desired spatial and temporal patterns.

### Generation of transgenic *Drosophila* lines

hPLD1 cDNA was amplified from the pCMV3-hPLD1 (a kind gift from Dr Nicholas Ktistakis). Not1 and Xba1 sites were used to amplify the amplicon of 3224 bp and ligated into pUAST-attB vector. Similar cloning strategy was used to amplify hPLD2 from pEGFPC2-hPLD2 (a kind gift from Prof Mike Wakelam) with Not1 and Xba1 restriction sites. GFP or mCHERRY tag was introduced in frame with hPLD2 using single-restriction site Not1 at the N-terminus of hPLD2. Transgenic animals with these constructs were generated using site-specific recombination-based transgenesis.

### Optical neutralization and scoring retinal degeneration

Flies were cooled on ice, decapitated using a sharp blade, and fixed on a glass slide using a drop of colorless nail varnish. Imaging was done using 40× oil objective of Olympus BX43 microscope. In order to obtain a quantitative index of degeneration, at least five flies were scored for each time point. A total of 50 ommatidia were assessed to generate degeneration index. To quantitate degeneration, a score of 1 was assigned to each rhabdomere that appeared to be wild-type. Thus, wild-type ommatidia will have a score of 7. Mutants undergoing degeneration will have a score between 1 and 7. Score were expressed as mean ± S.E.M.

### Immunohistochemistry for *Drosophila* photoreceptors

For immunofluorescence studies, retinae from flies were dissected under low red light in PBS. Retinae were fixed in 4% paraformaldehyde in PBS with 1 mg/ml saponin at room temperature for 30 min. Fixed samples were washed three times in PBTx (1× PBS + 0.3% Triton X-100) for 15 min each. The sample was then blocked in a blocking solution (5% FBS in PBTx) for 2 h at room temperature, after which the sample was incubated with primary antibody in blocking solution overnight at 4°C on a shaker. The following antibodies were used: rabbit anti-dPLD (1:500, Lab generated), rabbit anti-hPLD1K229-N term (1:200, kind gift from Dr Nicholas Ktistakis), and chicken anti-GFP (1:5000, Abcam [ab13970]). Appropriate secondary antibodies conjugated with a fluorophore were used at 1:300 dilutions [Alexa Fluor 488/568/633 IgG, (Molecular Probes)] and incubated for 4 h at room temperature. Wherever required, during the incubation with secondary antibody, Alexa Fluor 568-phalloidin (1:200, Invitrogen [A12380]) was also added to the tissues to stain the F-actin. After three washes in PBTx, sample was mounted in 70% glycerol in 1× PBS. Whole mounted preps were imaged on Olympus FV1000 confocal microscope using Plan-Apochromat 60×, NA 1.4 objective (Olympus).

### Immunohistochemistry for S2R^+^ cells

S2R^+^ cells stably expressing Actin-GAL4 were transfected using Effectene based as a transfection agent (Effectene Qiagen Kit [301425]). S2R^+^ cells were plated in 12-well plates; 0.5 μg of desired DNA construct was used for transfections. Forty-eight hours post transfection cells were allowed to adhere on glass cover-slip dishes for 1 h, fixed with 2.5% PFA in 1× M1 buffer for 20 min. They were washed and permeabilized with 0.37% Igepal (Sigma) in 1× M1 for 13 min. Blocking was performed in 1× M1 containing 5% FBS and 1 mg/ml BSA, for 1 h at RT. Then the cells were incubated with primary antibodies diluted in block solution for 2 h at RT, washed with 1× M1 several times, and incubated with appropriate secondary antibodies for 1–2 h at RT. These were then washed and imaged on an Olympus FV1000/FV3000 confocal microscope. The following antibodies were used: chicken anti-GFP(1:5000, Abcam [ab13970]), rabbit anti-hPLD1^N229^ (1:200, kind gift from Dr Nicholas Ktistakis), and rabbit anti-MYC (1:400, CST [2272S]). Appropriate secondary antibodies conjugated with a fluorophore were used at 1:300 dilutions [Alexa Fluor 488/568/633 IgG, (Molecular Probes)]. Alexa Fluor 488-phalloidin (1:400, Invitrogen [A12379]) was used to stain the F-actin (plasma membrane).

### Lipid extraction

Lipid extraction was performed using a modified Bligh and Dyer [31] method. For each sample, ten *Drosophila* heads were homogenized in 0.1 ml of 0.1 N ice-cold methanolic HCl and 30 μl of internal standard mixture. The internal standard mixture contained PA (17:0/14:1), PC (17:0/14:1), LPA (13:0), LPC (13:0), LPC (17:1), PA (17:0/17:0), PA (16:0-D31/18:1), LPA (17:1), LPC (19:0), PI (12:0/13:0), PA (12:0/13:0), and PC (12:0/13:0) and the concentrations of each of the standards were such that the final amount of any lipid standard falls within the linear response curve of the mass spectrometer. The linear response curve was determined to be between 1 femtomole and 12 picomole for PA standards. Tissues were homogenized using a Preceleys automated homogenizer that allows rapid and simultaneous treatment of all the samples in a given experiment. The methanolic homogenate was transferred into a 2-ml capped Eppendorf tube. Ice-cold 0.1 N methanolic HCl (0.2 ml) was used to recover any residual material in the homogenizer tube and was combined in the 2-ml Eppendorf tube. Further 0.1 ml of 0.1 N ice-cold methanolic HCl was added and then 0.8 ml chloroform was added and mixed thoroughly. This 1:2 methanol:chloroform mixture containing tissue homogenates was left to stand on ice for 10 min following which 0.88% KCl (0.4 ml) was added and vortexed for 30 s. This mixture was then centrifuged at 1000***g*** for 10 min at 4°C to separate the aqueous and organic phases. The lower organic phase containing lipids was taken out very carefully without mixing with the aqueous phase and transferred into a fresh Eppendorf tube. This lipid solution was dried in a vacuum evaporator and resuspended in 420 μl of 2:1 methanol:chloroform mixture for analysis.

### Establishment of ‘MS’ and ‘MS/MS’ database

To facilitate MS analysis, we have developed a theoretical platform that allows the calculation of neutral mass and prediction of m/z values for the most common adducts along with fragments that might arise during fragmentation of individual PA molecules. The generalized chemical formula of PA is C_m+3_H_2m−2n+5_O_8_P, where m is the total number of carbon atoms and n is the total number of double bonds in the two fatty acyl chains. Similarly, one can also derive the generalized chemical formula for other phospholipids and lyso-phospholipids. For example, in PA, the lyso form is C_m+3_H_2m−2n+7_O_7_P. Using these generalized chemical formulae, we calculated the precursor masses and individual fragment masses that could arise based on the different fatty acyl chain compositions at *sn−1* and *sn−*2. The Microsoft Excel workbook, named as ‘MS1_database.xlsx’ computes the lipid species formula, neutral mass, and m/z values for the nine most common adducts, [M+H]^+^, [M+H–H_2_O]^+^, [M+NH_4_]^+^, [M+Na]^+^, [M+K]^+^, [M+Li]^+^, [M–H]^−^, [M+HCO_2_]^−^, [M+CH_3_CO_2_]^−^ by taking into account, user-defined information of the total number of carbon atoms and total number of double bonds in the fatty acyl chains of PA. A mass of a single species of PA (e.g. 34:2) could be composed by a range of fatty acyl chains at *sn−1* and *sn−2* that vary in the number of carbon atoms and double bonds but add up to 34:2. To resolve this problem, we have generated a second workbook named as ‘MSMS_database.xlsx’ where we derive fragmentation masses corresponding to a specific lyso species by providing the number of carbon atoms and double bonds in a single fatty acyl chain. The experimental detection of a subset of these specific fragments can be used to establish the identity of the acyl chain at *sn−1* and *sn−*2. Collectively these Excel sheets (i.e., MS_database.xlsx and MSMS_database.xlsx) allow the user to obtain the accurate information about each precursor masses and individual fragmentation masses arising from different PA molecules containing distinct fatty acyl chains. These Excel workbooks are attached as Supplementary Materials (Supplementary table S1: SuppT1MS_database.xlsx and Supplementary table S2: SuppT2MSMS_database.xlsx) and also can be downloaded from http://flyfat.ncbs.res.in/resources. This approach can also be more generally applied to other phospholipid classes (PC, PI, PS, PG, PE) by simply including the heteroatoms of the class-specific head group for the class of lipid under analysis. We validated the output of these databases experimentally using chemically synthesized standards of PA (Supplementary Figure S1).

### LC-MS

#### Chromatographic conditions

Chromatographic separation was performed on a Phenomenex Luna Silica Column (1 mm × 150 mm, 3 µm) at room temperature in a Waters Acquity UPLC I Class. The autosampler injection volume was set to 6 µl and the eluent flow rate to 210 µl/min. After 5 min of equilibration with 100% eluent A [Hexane:IPA:100 mM AqNH_4_COOH (68:30:2)], eluent B [IPA:Hexane:100 mM AqNH_4_COOH (70:20:10)] was linearly increased to 30% over 5 min, then to 80% over 5 min, then to 100% over 5 min, and was held constant at 100% for 5 min. At last, the column was equilibrated for 9 min.

#### ESI-MRM-MS/MS operating conditions

A hybrid triple quadrupole ion-trap mass spectrometer (6500 QTRAP, ABSciex, Singapore) with a TurboIon source operating in negative ESI mode was used. System operation and data acquisition were controlled using Analyst 1.6.2. Source parameters were optimized using flow injection analysis of the internal standard mixture. Accordingly, the ion spray voltage was set to –4.5 kV and source temperature (TEM) to 450°C. The collision activated dissociation gas (CAD) was set at 3 psi and nitrogen was used as the collision gas. The nebulizer gas (GS1) was set to 24 psi, the auxiliary gas (GS2) to 21 psi, and the curtain gas (CUR) to 30 psi. Compound-dependent ion path parameters [declustering potential (DP) −42 V, entrance potential (EP) −6 V, and collision cell exit potential (CXP) −12 V] were optimized using continuous infusion of internal standard mixture solution. Full product spectra along with precursor→product MRM transitions with varying CE (12–45 eV) were recorded for fragmentation analysis using enhanced product ion (EPI) scanning function available in this mass spectrometer (Supplementary Figure S2). The MRM-triggered IDA-based EPI simultaneously record precursor ion-product ion scanning and ‘on the fly’ MS/MS acquisition. MRM experiments were performed with CE of 39 eV to gain high sensitivity (Supplementary Figure S2). If a specific molecule elutes from the chromatographic column at any time during the run, its specified MRM pair can be detected and recorded as we have limited the number of MRMs to maximum of 75 and dwell time of 30 ms to increase the duty cycle of the machine. Experimental tuning was used to decide the best ionization parameters for PA, as described in this section.

#### Data analysis

Identification and quantitation of lipid molecular species was performed using Analyst 1.6.2 (ABSciex, Singapore), PeakView 2.0 (ABSciex, Singapore) and MultiQuant 3.0.1 (ABSciex, Singapore). Briefly, EPI mode was used for qualitative identification of fatty acyl chains in PA molecules. EPI spectra were analyzed in PeakView software where individual molecular species with exact fatty acyl chain composition was identified. MultiQuant was used for determination of peaks in three dimensions (time, intensity, and m/z). Regular triple quadrupole-based MRM mode was used for quantitation of PA molecular species. Quantitation of naturally occurring lipid molecular species was performed utilizing two internal standards, one with lower number of carbon in fatty acyl chains (PA (12:0/13:0)) and another with higher number of carbon and unsaturation in fatty acyl chains (PA (17:0/14:1)). These standards helped us to rule out any variation due to acyl chain length and unsaturation. In pilot experiments, quantitation was attempted using these two independent standards and no significant difference was found. Results presented in the present study represent quantitation with PA (17:0/14:1) standard. Statistical calculations were performed using Microsoft Excel and GraphPad Prism 5.01. Unpaired two-tailed *t*tests were carried out.

### Phylogenetic analysis of PLD sequences

**Figure 1:**
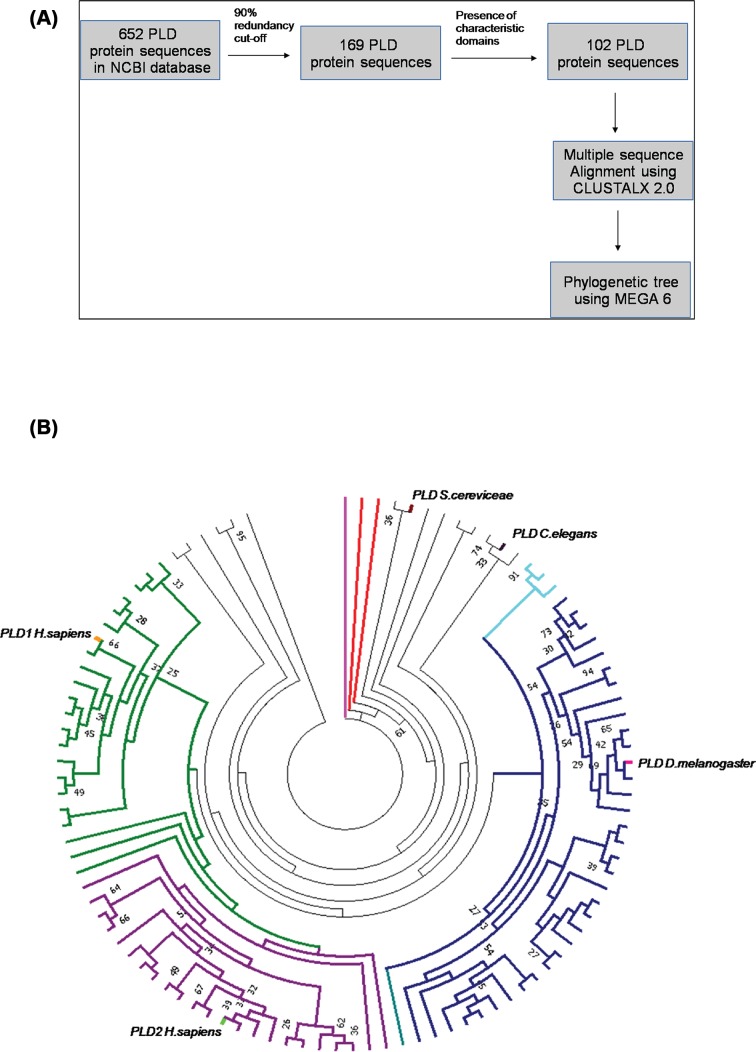
Phylogenetic analysis of phospholipase D sequences (**A**) Methodology – Workflow for phylogenetic analysis of PLD sequences. (**B**) Phylogeny of PLD sequences: the figure represents phylogenetic tree obtained using PLD protein sequences under study. The bootstrap values (>25) are marked for nodes obtained using the 50% consensus tree. The names of the taxa in clockwise order have been listed in Supplementary Table S3. Phospholipase A (PLA) sequence from *Rattus novergicus* has been used to root the tree (branch marked in magenta). Marked in red are PLDa and PLDb sequences from *Dictyostellium fasciculatum* (clockwise). PLD sequence from *S. cerevisiae and C. elegans* have been marked in maroon and thick black lines, respectively. The branches of the PLD sequences from class Insecta have been marked in dark blue and the PLD sequence of *Drosophila melanogaster* has been marked in pink. PLD sequences from class Mollusca (branches marked in cyan), PLD1 sequences from vertebrates (branches marked in green), and PLD2 sequences from vertebrates (branches marked in purple). hPLD1 and hPLD2 sequences have been labeled in orange and light green, respectively.

The protein sequences for PLD enzymes were obtained from the NCBI database. Six hundred and fifty-two sequences from organisms belonging to all kingdoms of life were collected; a cutoff at 90% sequence identity were used to discard identical sequences resulting in 169 sequences. For the present study, only sequences that had the intact domains characteristic of PLD enzymes (PLDc, PX, and the PH domains) were selected. PLD sequences from model organisms (*Saccharomyces cerevisiae, Arabidopsis thaliana*, and *Dictyostelium discoideum*) were retained irrespective of the above criteria. In subphylum Vertebrata, organisms wherein both PLD1 and PLD2 sequences were represented in the final set were selected. A final set of 102 PLD protein sequences was used for the phylogenetic analysis. Sequences were aligned using CLUSTALX ver. 2.0 [32]. The multiple sequence alignment thus generated was used as the input for the generation of a phylogenetic tree using the MEGA 6 tool (Figure 1A) [33]. The maximum likelihood method with 1000 bootstrap and JTT matrix was used to generate the phylogenetic tree. The clusters thus formed were analyzed based on the bootstrap values at each node in the tree.

## Results

### Phylogenetic analysis reveals that dPLD is evolutionarily similar to hPLD1

The PLD sequences selected as mentioned in ‘Experimental procedures’ and the phylogenetic tree with the maximum likelihood of occurrence was used for final interpretation of results. The tree at 50% consensus, i.e. clades clustered together more than 50% of the times in bootstrap replicates, is shown in Figure 1B and Supplementary table S3: SuppT3supplementary_table.xls.

The phospholipase A (PLA) sequence from *Rattus norvegicus*, used as an outlier, clusters at the base node. PLD sequences from the most primitive organisms (*Dictyostelium fasciculatum, Schistosoma* sp., *S. cerevisiae*, and *Hydra vulgaris*) form a distinct clade closer to the outlier. PLD sequences from plants (*A. thaliana*) and *Amphimedon queenslandica* form a clade indicating that they are divergent compared with PLD from invertebrates and chordates (Figure 1B). PLD sequences from molluscs and nematodes cluster together. In chordate genomes, we found two distinct genes encoding PLD proteins; these appear to be equivalent to that designated by prior cloning and experimental work in mammals as PLD1 and PLD2. Overall in our phylogenetic analysis, we noted three major subclusters of sequences namely insect PLD sequences (colored in navy blue), PLD1 (colored in green), and PLD2 (colored in purple) from chordates. Within the PLD1 and PLD2 subclusters, the PLD sequences from fish and amphibians cluster together as compared with those from reptiles, birds, and mammals. In most insect genomes, there appears to be a single gene encoding for a PLD-like protein. These insect PLD sequences appear to be evolutionarily closer to PLD1 sequences of chordates than to the PLD2 subcluster.

### Functional expression of hPLD1 and hPLD2 in *Drosophila* photoreceptors

**Figure 2:**
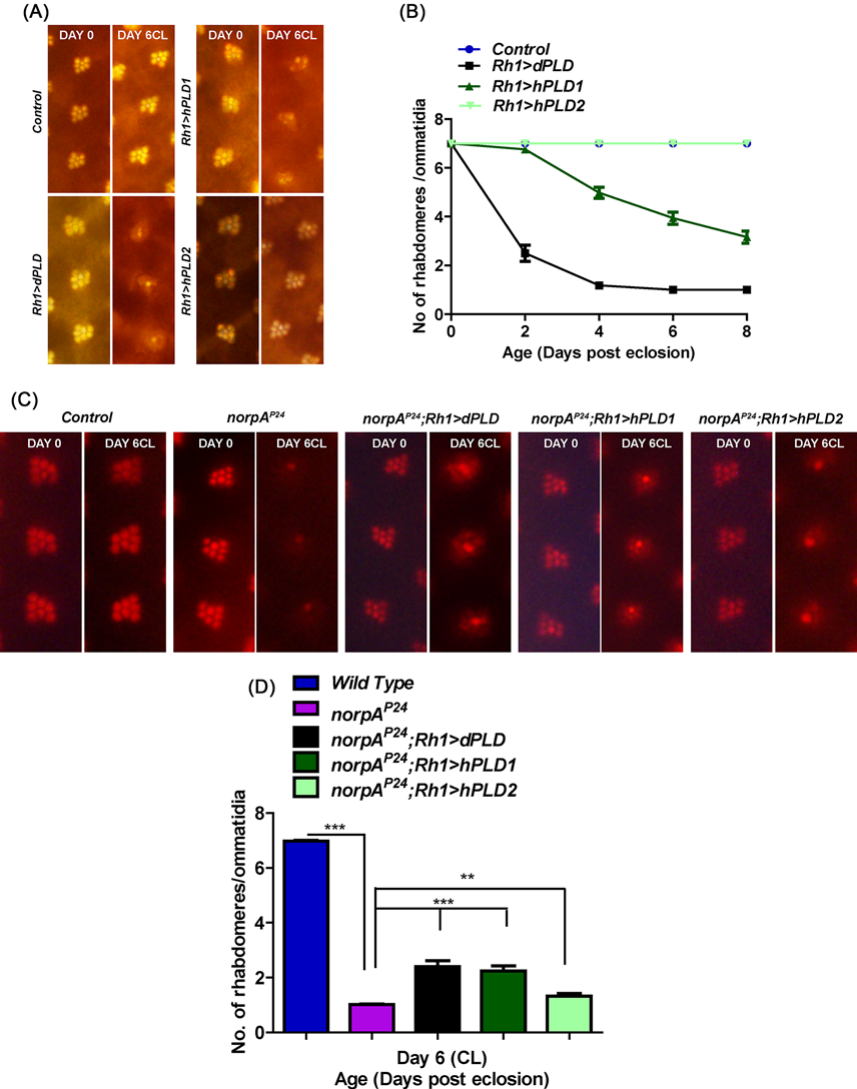
Functional analysis of human PLD genes in Drosophila photoreceptors (**A**) Representative optical neutralization (ON) images showing rhabdomere structure from control, Rh1 > dPLD, Rh1 > hPLD1, and Rh1 > GFP::hPLD2. The age and rearing conditions are mentioned on the top of the panels. (**B**) Quantitation of rate of photoreceptor (PR) degeneration of control, Rh1 > dPLD, Rh1 > hPLD1, and Rh1 > GFP::hPLD2 reared in bright light. The x-axis represents age of the flies and the y-axis represents the number of intact rhabdomeres visualized in each ommatidium. *n*=50 ommatidia taken from at least five separate flies. (**C**) Representative ON images showing rhabdomere structure from control, *norpA^P24^, norpA^P24^; Rh1 > dPLD, norpA^P24^; Rh1 > hPLD1*, and *norpA^P24^; Rh1 > GFP::hPLD2*. The age and rearing conditions are mentioned on the top of the panels. (**D**) Quantitation of rate of photoreceptor degeneration of control, *norpA^P24^, norpA^P24^; Rh1 > dPLD, norpA^P24^; Rh1 > hPLD1*, and *norpA^P24^; Rh1 > GFP::hPLD2* reared in bright light for 6 days. The x-axis represents age of the flies and the y-axis represents the number of intact rhabdomeres visualized in each ommatidium. *n*=50 ommatidia taken from at least five separate flies. Data were tested for statistics using unpaired *t*test. *** denotes *P*<0.001 and ** denotes *P*<0.01.

We generated transgenic *Drosophila* strains that allowed the expression of hPLD1 and hPLD2. These were expressed in adult *Drosophila* photoreceptors using the GAL4/UAS system; using the Rh1 promoter, we expressed hPLD1 and hPLD2 protein in these cells. The overexpression of dPLD in adult wild-type *Drosophila* photoreceptors (Rh1> dPLD) results in a progressive collapse of the apical domain of these cells, referred to as retinal degeneration, that is dependent both on time and illumination (Figure 2A,B). Likewise, we found that the overexpression of hPLD1 also resulted in light-dependent retinal degeneration although this was less severe than that seen by expression of dPLD. By contrast, the overexpression of hPLD2 did not result in any light-dependent retinal degeneration (Figure 2A,B). These findings suggest that hPLD1 can be expressed in *Drosophila* photoreceptors with functional consequences similar to that of dPLD expression.

It has previously been reported that overexpression of dPLD can partially rescue the retinal degeneration phenotype of *norpA* mutants (that lack PLCβ activity) by clearing Rhodopsin 1-loaded vesicles (RLVs) from the late endosomal compartment of the cell body of photoreceptors [29,34]. We tested the function of hPLD1 and hPLD2 by observing the consequences of overexpressing these proteins in *norpA^P24^* photoreceptors. As previously reported, *norpA^P24^* photoreceptors undergo retinal degeneration (Figure 2C,D) that was partially rescued by overexpression of dPLD (*norpA^P24^*; *Rh1 > dPLD*) (Figure 2C,D). We found that expression of hPLD1 (*Rh1 > hPLD1*) was able to suppress retinal degeneration in *norpA^P24^* as well as a dPLD transgene; however, expression of hPLD2 (*Rh1 > hPLD2*) was able to mediate only a minimal but statistically significant suppression of retinal degeneration in *norpA^P24^* (Figure 2C,D).

### hPLD1 is able to rescue the retinal degeneration of *dPLD^3.1^*

**Figure 3:**
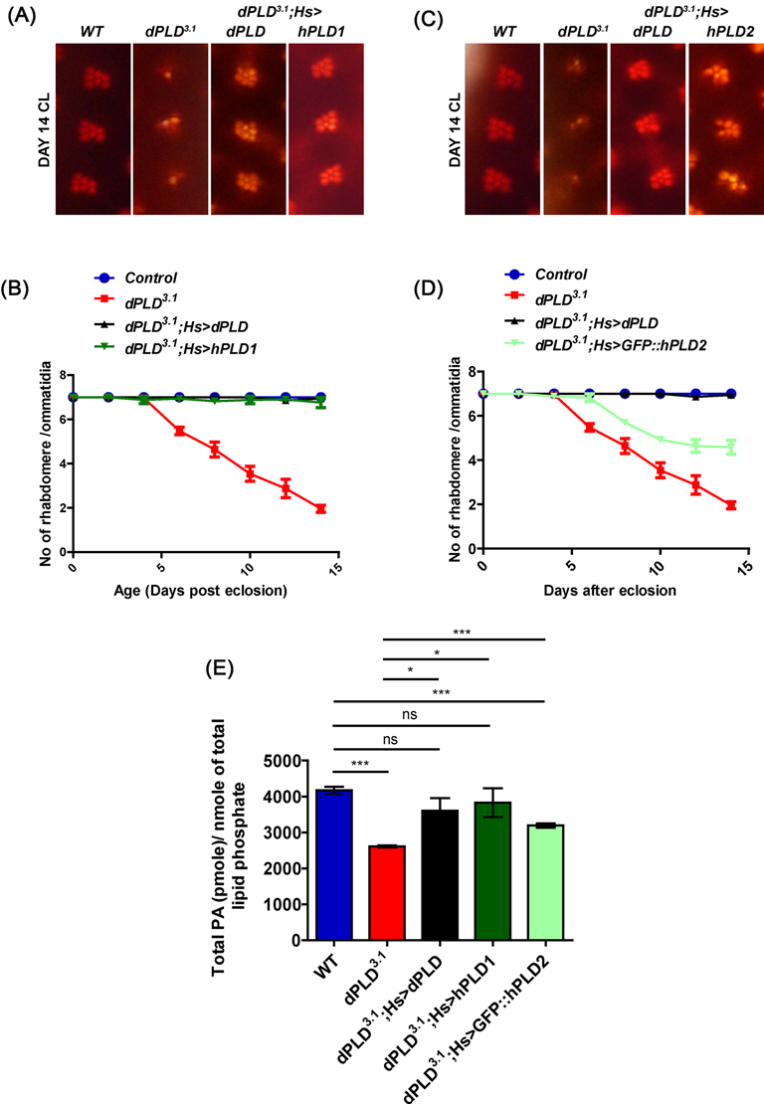
Functional and biochemical rescue of dPLD mutants by mammalian PLD genes Representative ON images showing ommatidia from (**A**) *wild-type (WT), dPLD^3.1^, dPLD^3.1^; Hs > dPLD, dPLD^3.1^; Hs > hPLD1*, and (**C**) *dPLD^3.1^; Hs> GFP::hPLD2*. The age and rearing conditions are indicated on the left side of the image. Quantitation of rate of PR degeneration of (**B**) *WT, dPLD^3.1^, dPLD^3.1^; Hs > dPLD, dPLD^3.1^; Hs > hPLD1*, and (**D**) *dPLD^3.1^; Hs > GFP::hPLD2* reared in bright light. *n*=50 ommatidia taken from at least five separate flies. (**E**) Total PA levels in head extract measured by normal phase LC-MRM-EPI-MS of *WT, dPLD^3.1^, dPLD^3.1^; Hs > dPLD, dPLD^3.1^; Hs > hPLD1*, and *dPLD^3.1^; Hs > GFP::hPLD2*. The x-axis represents the genotypes and the y-axis shows the level of total PA as pmole/nmole of total lipid phosphate present in the sample. Values are mean ± S.E.M., *n*=3. Unpaired *t*test was performed (*: *P*<0.05, ***: *P*<0.001, ns: not significant).

We have previously reported that a loss-of-function mutant in dPLD (*dPLD^3.1^*) undergoes progressive light-dependent retinal degeneration [29]. We tested the ability of the hPLD1 and hPLD2 to rescue this phenotype. When *dPLD^3.1^* was reconstituted with hPLD1 (*dPLD^3.1^; hs > hPLD1*), the light-dependent retinal degeneration was completely suppressed (Figure 3A,B). In contrast, when *dPLD^3.1^* was reconstituted with hPLD2 (*dPLD^3.1^; hs > hPLD2*), retinal degeneration was only partially suppressed (Figure 3C,D). These findings suggest that hPLD1 is able to functionally better substitute for the loss of dPLD than hPLD2.

To understand the biochemical basis of why hPLD1 is able to suppress retinal degeneration better than hPLD2, we measured PA levels using MS from head extracts of flies in which *dPLD^3.1^* was reconstituted with dPLD, hPLD1, or hPLD2. As previously reported [29], we found that reconstitution of *dPLD^3.1^*with dPLD (*dPLD^3.1^; hs > dPLD*) was able to restore the reduced PA levels seen in this mutant (Figure 3E). Likewise, in *dPLD^3.1^; hs > hPLD1* PA levels were restored to that of wild-type animals (Figure 3E). However, in *dPLD^3.1^; hs > hPLD2* extracts, although PA levels were higher than in *dPLD^3.1^*, they were not restored to the levels seen in wild-type flies (Figure 3E). Thus, while both hPLD1 and hPLD2 were able to restore the reduced PA levels in *dPLD^3.1^*, in the case of hPLD2 the restoration of total PA to wild-type levels was not complete.

### Subcellular localization of hPLD isoforms in *Drosophila* cells

**Figure 4:**
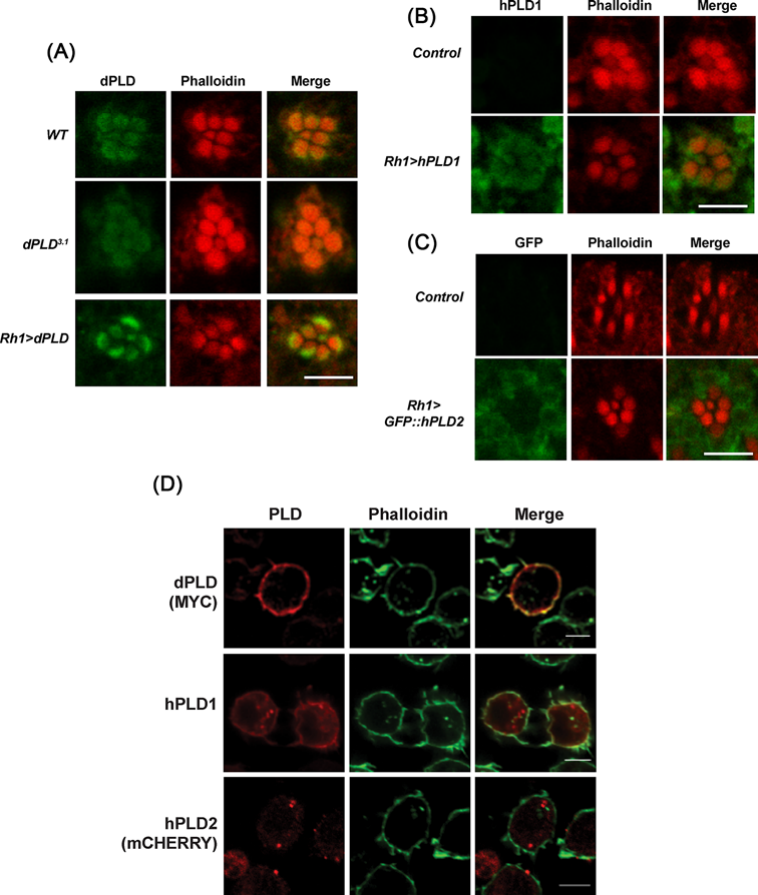
Sub-cellular localization of human PLD proteins in Drosophila cells (**A**) Transverse section (TS) of retinae from *WT, dPLD^3.1^*, and *Rh1 > dPLD* stained with dPLD antibody. Flies were dissected after 0–6 h (day 0) post eclosion. Scale bar: 5 µm. (**B**) TS of retinae from *Control* and *Rh1 > hPLD1* stained with hPLD1 antibody. Flies were dissected after 0–6 h (day 0) post eclosion. Scale bar: 5 µm. (**C**) TS of retinae from *Control* and *Rh1 > GFP::hPLD2* stained with GFP antibody. Flies were dissected after 0–6 h (day 0) post eclosion. Scale bar: 5 µm. (**D**) Confocal imaging showing co-localization of MYC::dPLD (stained with MYC), hPLD1 (stained with hPLD1 antibody raised against N-terminus of the protein) and mCHERRY::hPLD2 (stained for mCHERRY) in S2R^+^ cells with Alexa Fluor 488-phalloidin (Invitrogen). Scale bar: 5 µm.

We compared the localization of dPLD, hPLD1, and hPLD2 when reconstituted in *dPLD^3.1^* photoreceptors. Using an antibody raised against dPLD, we found that as previously reported [7,34], the protein is mainly localized to a region at the base of the microvillar plasma membrane and overexpressed dPLD remains restricted to this location as well (Figure 4A). Likewise, when hPLD1 was reconstituted in *dPLD^3.1^*, we found that it too showed localization to the base of the microvilli (Figure 4B); by contrast hPLD2 expressed in *dPLD^3.1^* photoreceptors was not found at the base of the microvilli but was distributed throughout the cell body at low levels (Figure 4C).

We also studied the localization of dPLD, hPLD1, and hPLD2 when expressed in *Drosophila* S2R^+^ cells. When dPLD was expressed in S2 cells, it co-localized to a number of subcellular compartments including Rab5 and Rab7 positive structures (Supplementary Figure S3A) and showed some co-localization with subplasma membrane actin domain (Figure 4D). When hPLD1 was expressed in S2 cells, it was found in punctate intracellular structures where it co-localized with Rab5, mannosidase (Supplementary Figure S3B) and also the subplasma membrane actin domain (Figure 4D). In contrast, hPLD2 co-localized with Rab5, Rab7, and mannosidase in punctate structures (Supplementary Figure S3C) but no localization was detected in the subplasma membrane actin domain (Figure 4D). Thus, hPLD1 and hPLD2 show differences in subcellular localization when expressed in *Drosophila* cells.

### Developing a method to determine the acyl chain composition of PA species

**Figure 5:**
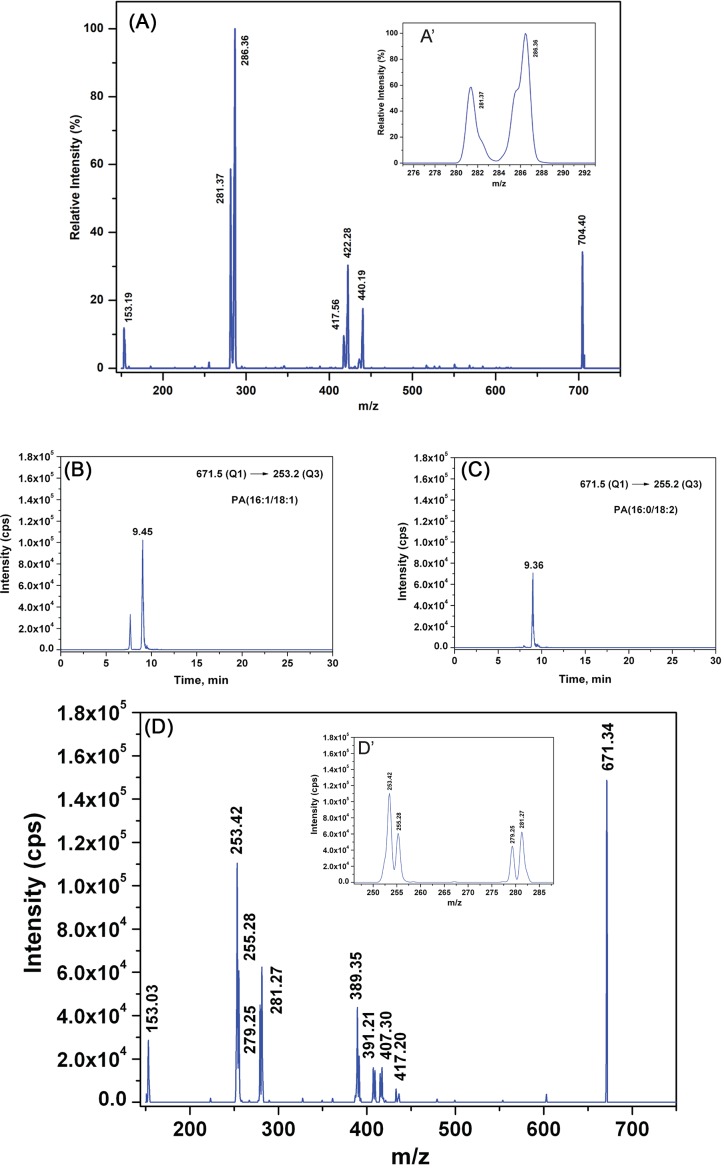
Identification of the fatty acyl chain composition of PA by mass spectrometry EPI spectra of (**A**) precursor mass of 704.40 of synthetic PA (16:0D31/18:1). (**A’**) The resulting *sn−1* fatty acid carboxylate ion and *sn−2* counterpart are shown in the inset. (**B**) XIC of 671.34–253.2 MRM transition and (**C**) of 671.4–255.2 MRM transition from *Drosophila* head lipid extract. The ion count of the corresponding transitions are represented (y-axis) with reference to the elution time of the molecules from the LC column (x-axis). (**D**) EPI spectra of precursor mass of 671.34 from *Drosophila* head lipid extract. (**D’**) Fatty acid carboxylate ions observed are as follows: m/z 253.42, 255.28, 279.25, 281.27, and relative levels of these masses are shown in the inset.

Like most other lipid classes, PA is present in cells as a complex mixture of molecular species that show diversity in the acyl chain length and unsaturation of the fatty acyl chain at sn−1 and sn−2 positions [35]. To understand the role of acyl chain diversity, if any, in the PA generated by PLD1 and PLD2, we developed a method to establish the definitive acyl chain composition of PA species. We investigated whether differential fragmentation patterns obtained from MRM-triggered information dependent (IDA) ‘on the fly’ MS/MS’ (recorded as EPI) experiments, could be used diagnostically to establish *sn−1* and *sn−2* positional isomers present in PA from biological samples. The representative tandem mass spectra of synthesized, *sn−1* chain pre-deuterated 16:0D31/18:1-PA recorded at collision energy of 39 eV in EPI scan mode is shown (Figure 5A). We found that the deuterated acyl chain in *sn−1* position gave rise to the peak with highest intensity at m/z 286.36 (255.36 for 16:0 acyl anion+31 for heavy isotope of hydrogen) ([R_1_COO]^−^), followed by second highest peak at m/z 281.37 corresponding to *sn−2*, 18:1 non-deuterated fatty acyl anion ([R_2_COO]^−^) (Figure 5A’). These observations are in agreement with the previously reported study by Hsu and Turk [36] that used 16:0/18:1-PA standards over a smaller collision energy range of 25–35 eV. We coupled LC separation of a complex lipid mixture (from *Drosophila* head extracts) with the mass spectrometer as a detector and employed MRM as well as MRM-triggered EPI scans. Using the database described in the ‘Experimental procedures’ section (MS_database.xlsx and MSMS_database.xlsx), we selected ions corresponding to the deprotonated form ([M–H]^−^) of PA in the first quadrupole and one of its constituent fatty acids in the third quadrupole of the mass spectrometer. In the MRM-triggered EPI scans; we identified the product ions. Using this approach, a stepwise testing of all the precursor/product ion pairs (MRM transitions) predicted in the database were performed on *Drosophila* lipid extracts. The MRM-Ion pair list of all the molecular ions and their fragment ions tested in the present study is given in the Microsoft Excel workbook-‘Combined_PA_database.xlsx’ as Supplementary table S4: SuppT4Combined_PA_database.xlsx and can also be found at http://flyfat.ncbs.res.in/database. A series of representative molecules of precursor masses 691.28, 693.23, 695, 697.30, and 699.33 and the MS/MS fragmentation masses arising out of them in EPI scans are shown in Supplementary Figure S4. Based on these observations we conclude that different molecular species of PA can be identified with exact composition of the *sn−1* and *sn−2* fatty acid positional isomers. Most importantly, the relative intensity of a molecular species derived from only MRM transition-based experiments is very well correlated with that found in the MRM-triggered IDA-based EPI scans. As an illustrative example, in case of precursor ion of 671.34, which is PA[34:2] (from HRMS measurement) [29], the corresponding MRM transitions that need to be checked are (i) 671.46–255.20 (16:0), (ii) 671.46–279.23 (18:2), (iii) 671.46–251.20 (16:2), (iv) 671.46–283.26 (18:0), (v) 671.46–253.21 (16:1), (vi) 671.46–281.24 (18:1), (vii) 671.46–223.17 (14:2), and (viii) 671.46–311.29 (20:0). We observed that 671.46–223.17 (14:2) transition and 671.46–311.29 (20:0) transition are absent. As shown in Figure 5D and inset therein (Figure 5D’), the relative intensity profile of fatty acid fragments as observed from the spectra is as follows – I_253.42_ > I_255.28_ = I_281.27_ > I_279.25_. From this intensity profile, we conclude that there are two specific molecules within PA[34:2] and the exact position of these fatty acyl components will be 16:1 in *sn−1* with 18:1 in *sn−2* (I_253.42_ > I_255.28_ = I_281.27_), 16:0 in *sn−1* with 18:2 in *sn−2* (I_255.28_ = I_281.27_ > I_279.25_). This arrangement of fatty acyl chains has further been verified by the retention time of the individual MRM transitions. The highest peak amongst all the transitions is from PA(16:1/18:1) whose retention time is 9.45 min as seen from extracted ion chromatogram (XIC) in Figure 5B. The next highest arises from PA(16:0/18:2) with retention time of 9.36 min (Figure 5C).

### hPLD1 and hPLD2 produce distinct molecular species of PA

**Figure 6:**
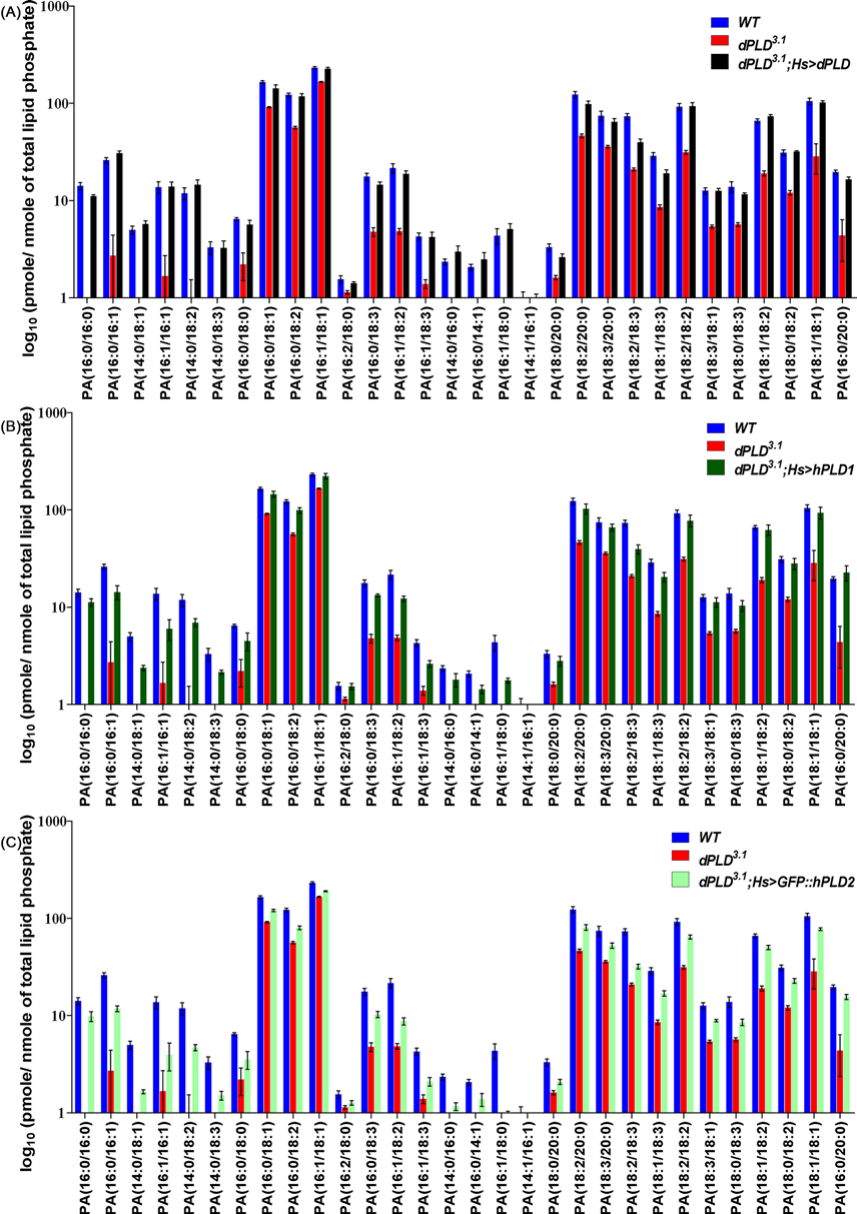
Quantification of individual molecular species of PA from Drosophila heads Relative changes in different PA molecular species across different genetic manipulations are presented. x-axis shows different PA molecular species. y-axis shows level of PA molecular species as log_10_ (pmole per nmole of total organic phosphate). A comparison of the level of each molecular species between loss-of-function mutant in dPLD (dPLD^3.1^) and wild-type control with (**A**) reconstitution with dPLD (*dPLD^3.1^; hs > dPLD*), (**B**) reconstitution with hPLD1 (*dPLD^3.1^; hs > hPLD1*), and (**C**) reconstitution with hPLD2 (*dPLD^3.1^; hs > hPLD2*) are presented.

Using the above method, we identified and quantitated 39 unique molecular species of PA in lipid extracts from *Drosophila* heads. We found that the levels of 30 of these PA species were lower in *dPLD^3.1^* compared with controls (Figure 6A). When *dPLD^3.1^*was reconstituted with dPLD (*dPLD^3.1^; hs > dPLD*), the levels of 26 of these species were restored to wild-type levels (Figure 6A and Supplementary Table S5). In the case of *dPLD^3.1^*; hs > hPLD1, 16 species were restored to wild-type levels (Figure 6B and Supplementary Table S5). By contrast, in *dPLD^3.1^*; *hs > hPLD2*, only one species namely 16:2/18:0 could be restored to wild-type levels (Figure 6C and Supplementary Table S5). Amongst the species that were not restored to wild-type levels, the levels of several were substantially elevated above that seen in *dPLD^3.1^*. We also tested the ability of dPLD, hPLD1, and hPLD2 to elevate the levels of each species above that seen in *dPLD^3.1^*. When the data were analyzed from this perspective, we found that dPLD was able to significantly elevate the level of all PA species over that seen in *dPLD^3.1^*; hPLD1 was unable to elevate the levels of two species (Figure 6B and Supplementary Table S5) and hPLD2 unable to elevate the levels of five species of PA (Figure 6C and Supplementary Table S5). Thus, when expressed in *Drosophila* cells lacking PLD activity, hPLD1 and hPLD2 were both able to generate a range of PA species but differed in their effectiveness in elevating the levels of defined molecular species and restoring their levels to wild-type flies.

## Discussion

In vertebrates, two conventional PLD genes with demonstrated enzyme activity namely PLD1 and PLD2 have been studied. Both hPLD1 and hPLD2 are widely expressed with transcripts being found in numerous cell types and in a broad range of tissues as reported in multiple human tissue expression catalogs that have been recently collected [37–39]. Likewise, in organisms with a smaller and less complex genome such as *Drosophila*, conventional PLD activity is encoded by a single gene that is widely expressed across tissues and during development. Thus, it is unlikely that PLD1 and PLD2 represent two genes encoding polypeptides with the same enzymatic activity but with differential expression patterns across various vertebrate tissues. Through a detailed phylogenetic analysis, we found that PLD1 and PLD2 sequences form distinct clades and the PLD1 clade (including hPLD1) is in fact more closely related to the single PLD gene seen in the genomes of insects; the PLD2 clade (including hPLD2) seems more distantly related to the single insect PLD gene in insects. At the level of protein sequence, all major domains of PLD proteins such as the PX, PH, and HKD domains are conserved across clades with much of the sequence variation arising from the inter-domain sequences in the two polypeptides. Thus, it is apparent from these analyses that hPLD1 is perhaps equivalent to the single PLD seen in insects. Interestingly the PLD1 and PLD2 genes in mouse are unusually different in size; while PLD1 is 147 kb, PLD2 is 17.1 kb in size [40]. Thus, the evolution of the second PLD gene must have involved gene duplication followed by either the loss of a large amount of DNA sequence (if hPLD2 evolved later) or the gain of DNA sequence (if hPLD1 evolved later).

To understand the potential functional differences between hPLD1 and hPLD2, we tested their ability to revert phenotypes of a null mutant in the single *Drosophila* PLD gene (*dPLD^3.1^*). Briefly, we found that (i) hPLD1 was able to completely rescue the retinal degeneration phenotype of *dPLD^3.1^*, while hPLD2 was able to affect only a partial rescue (ii) hPLD1 was able to suppress the retinal degeneration phenotype of *norpA^P24^* to an extent equivalent to dPLD whereas hPLD2 was less effective in this regard. (iii) Finally, when overexpressed in wild-type photoreceptors hPLD1 was able to elicit a stronger light-dependent retinal degeneration than hPLD2. Together, these observations support the conclusion that hPLD1 is able to functionally substitute better for dPLD than hPLD2 in *Drosophila* cells. This conclusion is also consistent with our phylogenetic analysis indicating that hPLD1 is more closely related to dPLD than hPLD2.

Why is hPLD1 able to functionally substitute better for dPLD than hPLD2? One possibility is the lower levels of hPLD2 protein expression. Although hPLD2 was expressed at lower protein levels than hPLD1, both were able to generate large rises of total PA levels when reconstituted in cells lacking dPLD (Figure 3E), suggesting that they are both substantially active when expressed in *Drosophila* cells. This observation is consistent with previous studies in mammalian cells demonstrating higher basal and stimulated specific activity for hPLD2 compared with hPLD1 [41]. Thus, lower protein expression is unlikely to explain the reduced effectiveness of hPLD2 compared with hPLD1 in rescuing retinal degeneration in *dPLD^3.1^*.

A second possibility is that although hPLD1 and hPLD2 are biochemically equally active, they are localized at distinct subcellular compartments. It is reported that in *Drosophila* photoreceptors dPLD localize to a region close to the plasma membrane at the base of the microvilli [7,34]. At this location, the plasma membrane also forms a membrane contact site with the submicrovillar cisternae (SMC) (reviewed in [42]). Presently it is unclear if dPLD is localized to the membrane of the SMC or to the microvillar plasma membrane in this area; immunogold labeling studies with TEM will be required to establish this point. When expressed in photoreceptors hPLD1 also localizes partially to the region of the SMC/plasma membrane contact site whereas hPLD2 was excluded from this region. In independent experiments where hPLD1 and hPLD2 were expressed in S2 cells, we found that while dPLD and hPLD1 show some degree of localization with subplasma membrane actin, no such localization is seen for hPLD2. Conceptually, this may reflect a subcellular localization to plasma membrane-ER contact sites similar to that seen in photoreceptors. Although hPLD1 and hPLD2 also showed variable localization to a number of other vesicular compartments, the ability to localize to a subplasma membrane compartment in photoreceptors may represent a conserved feature of dPLD and hPLD1 allowing them to affect a rescue of *dPLD^3.1^*phenotypes.

In cells, lipid classes such as PC show remarkable molecular heterogeneity with a large range of variations in the acyl chain length and unsaturation of the individual fatty acids [43]. Although hPLD1 and hPLD2 both generate PA using the substrate PC, it was possible that each of these two enzymes might have a preference for the acyl chain length and unsaturation of the PC that they can use as substrate. Consequently, the PA that they produce could also have a unique acyl chain composition and the variable biological activity of such molecular species might explain the separate requirement for hPLD1 and hPLD2. For example, it has been previously proposed that PA species produced by the activity of diacylglycerol kinase and PLD might have non-overlapping acyl chain composition and may contribute to function [44] although a lipidomics analysis of brain tissue from wild-type and PLD2^−/−^ mice has not demonstrated the ability of PLD2 to produce specific molecular species of PA [14]. To test the possibility that the differential ability of hPLD1 and hPLD2 to rescue *dPLD^3.1^* arises from their production of distinct molecular profiles of PA species, we developed an analytical method to accurately determine the acyl chain composition of the fatty acids at the *sn−1* and *sn−2* position of PA. Using this method, we found that 30 of the 39 detectable species of PA showed reduced levels in *dPLD^3.1^* and the levels of all but four of these species were completely restored on reconstitution with dPLD. However, when *dPLD^3.1^* was reconstituted with hPLD1 and hPLD2, in each case, the levels of a subset of molecular species could not be rescued to wild-type levels. While hPLD1 reconstitution restored 16 species of PA to wild-type levels, hPLD2 reconstitution restored only 1 species to wild-type levels. Conversely, while hPLD1 reconstitution failed to elevate the levels of only two PA species above that in *dPLD^3.1^*, in case of hPLD2 reconstitution five species remained the same as in *dPLD^3.1^*. It is possible that those species of PA whose levels could be restored to wild-type levels by hPLD1 but not hPLD2 may be mediators of the effects of PA in tuning apical membrane turnover in *Drosophila* photoreceptors; additional experiments will be needed to test this hypothesis. However, it must also be noted that to date specific biological functions for molecular species of PA with unique acyl chain length have not been described.

In summary, our studies suggest that of the two conventional isoforms in mammalian genomes hPLD1 seems most closely related to dPLD in terms of sequence and subcellular localization and the ability to better substitute for dPLD in our assays. By contrast, hPLD2 shows a distinct subcellular localization and may have evolved as an enzyme to generate PA at distinct subcellular compartment membranes and mediate alternate molecular processes in these locations. The subcellular localization of endogenous PLD1 and PLD2 in mammalian cells remains to be determined unequivocally and will contribute to understanding the function of these isoforms.

## Supporting information

**Table:** 

**Table:** 

**Table:** 

**Table:** 

**Table:** 

## Abbreviations

EPI: enhanced product ion
HKD: HxKxxxxDx6 GSxN motif
hPLD1: human PLD1
hPLD2: human PLD2
PA: phosphatidic acid
PBTx: 1× PBS + 0.3% Triton X-100
PC: phosphatidylcholine
PLD: phosphatidylcholine-specific phospholipase D
SMC: submicrovillar cisternae
