# Supplementary material for: Functional analysis of mammalian phospholipase D enzymes
Source: Biosci Rep. 2018 Dec 7;38(6):BSR20181690. doi: 10.1042/BSR20181690 (PMC6435507; doi:10.1042/BSR20181690)
Supplement: Supplementary file 5 [file bsr20181690_Supp5.pdf]

Compared to WT					Compared to PLD3.1		
Molecular Species	dPLD	hPLD1	hPLD2		dPLD	hPLD1	hPLD2
PA(16:0/16:0)	0.036	0.0957	0.026		0.000	0.000	0.000
PA(16:0/16:1)	0.076	0.0023	0.000		0.000	0.003	0.001
PA(14:0/18:1)	0.309	0.0005	0.000		0.000	0.000	0.003
PA(16:1/16:1)	0.955	0.0096	0.002		0.000	0.037	0.194
PA(14:0/18:2)	0.317	0.0223	0.002		0.000	0.000	0.000
PA(14:0/18:3)	0.947	0.0435	0.006		0.001	0.000	0.000
PA(16:0/18:0)	0.264	0.0692	0.004		0.004	0.078	0.215
PA(16:0/18:1)	0.126	0.1283	0.000		0.002	0.001	0.000
PA(16:0/18:2)	0.681	0.0217	0.000		0.000	0.000	0.000
PA(16:1/18:1)	0.577	0.5463	0.000		0.000	0.003	0.000
PA(16:2/18:0)	0.362	0.9433	0.095		0.003	0.010	0.142
PA(16:0/18:3)	0.109	0.0172	0.001		0.000	0.000	0.000
PA(16:1/18:2)	0.340	0.0045	0.001		0.000	0.000	0.001
PA(16:1/18:3)	0.919	0.0028	0.000		0.000	0.001	0.020
PA(14:0/16:0)	0.210	0.1214	0.000		0.000	0.001	0.000
PA(16:0/14:1)	0.362	0.0121	0.024		0.001	0.002	0.006
PA(16:1/18:0	0.510	0.0105	0.002		0.000	0.006	0.256
PA(14:1/16:1)	0.931	0.0009	0.006		0.002	0.148	0.961
PA(18:0/20:0)	0.078	0.2644	0.003		0.002	0.005	0.009
PA(18:2/20:0)	0.063	0.2188	0.003		0.000	0.001	0.000
PA(18:3/20:0)	0.337	0.4220	0.035		0.000	0.000	0.001
PA(18:2/18:3)	0.000	0.0006	0.000		0.000	0.002	0.000
PA(18:1/18:3)	0.007	0.0270	0.001		0.000	0.000	0.000
PA(18:2/18:2)	0.933	0.2898	0.006		0.000	0.002	0.000
PA(18:3/18:1)	0.983	0.4195	0.004		0.000	0.001	0.000
PA(18:0/18:3)	0.237	0.1413	0.017		0.000	0.005	0.002
PA(18:1/18:2)	0.145	0.6570	0.002		0.000	0.000	0.000
PA(18:0/18:2)	0.699	0.4814	0.006		0.000	0.002	0.000
PA(18:1/18:1)	0.739	0.4701	0.007		0.000	0.003	0.001
PA(16:0/20:0)	0.046	0.4822	0.013		0.000	0.002	0.000
		p>0.05					p>0.05
	Not lower than Wild Type					Not elevated above dPLD3.1	
